# Sportmedizinische Langzeitbetreuung von Soldaten: Bedeutung von Rumpfkraft und Leistungsfähigkeit

**DOI:** 10.1007/s00132-025-04677-w

**Published:** 2025-07-23

**Authors:** Christoph Schulze, Sabrina Fehrmann, Claudia Bünzen, Daniel Geißler

**Affiliations:** 1 Zentrum für Sportmedizin der Bundeswehr, Warendorf, Deutschland; 2 Universitätsinstitut für Physikalische Medizin und allgemeine Rehabilitation, Universitätsklinikum Salzburg, Paracelsus Medical University, Müllner-Hauptstraße 48, 5022 Salzburg, Österreich; 3 Orthopädische Klinik und Poliklinik, Universitätsmedizin Rostock, Doberaner Str. 142, 18057 Rostock, Deutschland; 4 Bundeswehrkrankenhaus Hamburg Klinik III, Hamburg, Deutschland; 5 Institut für Sportmedizin und Trainingswissenschaft, Christian-Albrechts-Universität Kiel, Kiel, Deutschland; 6 Klinik für Orthopädie und Unfallchirurgie, Septisch-Rekonstruktive Chirurgie, Bundeswehrkrankenhaus Berlin, Berlin, Deutschland

**Keywords:** Ergometrie, Fettstoffwechsel, Muskelkraft, Körperliche Leistungsfähigkeit, Primärprävention, Ergometry, Lipid metabolism, Muscle strength, Physical performance, Primary prevention

## Abstract

**Hintergrund:**

Soldaten sind starker körperlicher Beanspruchung mit einem erhöhten Risiko zur Entwicklung von Überlastungsschäden ausgesetzt. Maßnahmen zum Erhalt der körperlichen Leistungsfähigkeit haben große präventive Bedeutung.

**Fragestellung:**

Kann eine sportmedizinische Langzeitbetreuung zum Erhalt der körperlichen Leistungsfähigkeit beitragen?

**Material und Methoden:**

Retrospektiv wurden bei 115 Soldaten über 6 Jahre sportmedizinische Untersuchungsparameter aus isokinetischen Rumpfkraftmessungen, Lactat-Leistungsdiagnostik, 50-m-Sprinttest, Anthropometrie und Laborparametern evaluiert und mittels ANOVA und Pearson-Korrelationsanalyse ausgewertet.

**Ergebnisse:**

Es zeigten sich keine relevanten Veränderungen bei anthropometrischen Parametern. HDL-Cholesterin und Rumpfextensionskraft stiegen signifikant an. Die Leistung im Sprinttest verbesserte sich signifikant. Die maximale Geschwindigkeit in der Leistungsdiagnostik blieb konstant.

**Schlussfolgerungen:**

Eine sportmedizinische Langzeitbetreuung von Soldaten ist zur Prävention eines Leistungsverlustes geeignet und bietet Potenzial zur Verbesserung der Performance.

## Abstract (en)

**Background:**

Soldiers are exposed to physical strain with increased risk of developing overload injuries. Measures to preserve physical performance are of great preventive importance.

**Purpose:**

Does a long-term sports medical support based on strength and performance parameters help to maintain physical performance capacity?

**Methods:**

Data from isokinetic trunk strength measurements, lactate performance diagnostics, 50 m sprint tests, as well as anthropometric and laboratory parameters were retrospectively evaluated in 115 soldiers over 6 years and analysed using ANOVA and Pearson correlation analysis.

**Results:**

There were no relevant changes in anthropometric parameters. HDL cholesterol and trunk extension strength increased significantly. Performance in the sprint test also improved significantly. The maximum speed in the performance diagnostics remained constant.

**Conclusion:**

Long-term sports medical care for soldiers is suitable for preventing a loss of performance capacity and offers potential for further improvement.

Die Prävention von Überlastungsschäden am Bewegungsapparat und der Erhalt der Einsatzfähigkeit sind für Soldaten bedeutsam und stehen in einem direkten Zusammenhang. Für die Feststellung des Erhalts der Leistungsfähigkeit eignen sich sportmedizinische Untersuchungen mit Analyse von Rumpfkraft und kardiorespiratorischer Belastbarkeit. Die Bedeutung des Monitorings, aus orthopädischer Sicht, insbesondere der Rumpfkraft, soll in dieser Arbeit dargestellt werden.

## Einleitung

Soldaten werden während des Dienstes starken körperlichen Beanspruchungen ausgesetzt [1]. Es besteht dabei ein erhöhtes Risiko für das Auftreten von Verletzungen und Überlastungsschäden, wie Marschfrakturen und Rückenschmerzen mit der möglichen Folge längerer Krankheitsphasen, die die Einsatzbereitschaft, insbesondere von körperlich hochbelasteten Einheiten, gefährden können [2, 3]. In Abhängigkeit von der Verwendung wurde auch das Risiko für eine Gewichtszunahme und die Entwicklung von Risikofaktoren für eine Einschränkung der kardiopulmonalen Leistungsfähigkeit beobachtet [4, 5]. Um eine bessere Resilienz gegenüber körperlichen Beanspruchungen am Arbeitsplatz zu erreichen, eignen sich den Erfordernissen der Tätigkeit angepasste Trainingsmaßnahmen [6].

Bei Leistungssportlern wurden zur Überprüfung der Sport- und Wettkampftauglichkeit zusätzlich Kaderuntersuchungen eingeführt. Hierbei besteht, ergänzend zum Training, die Möglichkeit einer professionellen Beratung unter Berücksichtigung sportmedizinischer Aspekte zur Vermeidung von Übertraining und daraus resultierenden Überlastungsschäden [7]. In der Sportlerbetreuung werden zusätzlich Trainingsbereiche für das Ausdauertraining ermittelt, aber auch Empfehlungen zur Durchführung von Krafttraining gegeben. Kraft und Ausdauer sind auch Grundlagen soldatischen Trainings [8].

Leistungssportler unterliegen häufig einem starken wirtschaftlichen Druck, der unmittelbar mit dem Erhalt ihrer körperlichen Leistungsfähigkeit verbunden ist [9]. Da die Ausbildung von Soldaten in speziellen Verwendungen ebenfalls teuer und zeitaufwendig ist, wird die Prävention von Verletzungen, Überlastungsschäden und des Verlusts der körperlichen Leistungsfähigkeit bei diesem Personenkreis als wichtige medizinische Unterstützungsleistung angesehen [10]. Eine Kombination aus „Kaderuntersuchung“ zur Feststellung gesundheitlicher Probleme, aber auch die Möglichkeit zur Optimierung des individuellen Trainings, sollte dabei abgebildet werden. Eine Ermittlung des Vorhandenseins von Risikofaktoren sowie die Evaluation der Ausdauerleistungsfähigkeit, der Sprintfähigkeit, aber auch der Rumpfkraft wurde hierfür als notwendig erachtet. Die Bedeutung der Rumpfkraft liegt für Soldaten insbesondere darin begründet, dass sie häufig schwere Lasten, zum Teil über längere Strecken, tragen müssen. Dies ist einhergehend mit einem erhöhten Risiko für das Auftreten von Rückenschmerzen [11]. Andere Autoren berichteten, dass die Rumpfkraft für die Entwicklung von höherer Belastbarkeit gegenüber sportlicher Beanspruchung, aber auch im Rahmen der Entwicklung von Leistungsfähigkeit von Bedeutung ist [12, 13]. Eine valide Methode für die Messung der Rumpfkraft stellt dabei deren isokinetische Bestimmung dar [14]. Diese wird durch anthropometrische Faktoren spezifisch beeinflusst und es wurden Verbindungen zur Entwicklung von anderen Leistungsparametern nachgewiesen, was die Anwendung auch im präventiven Setting sinnvoll macht [8]. Außerdem waren bei den zu betreuenden Soldaten berufs- und sportartspezifische Besonderheiten bekannt, die bei Trainingsempfehlungen berücksichtigt werden konnten [15]. Dass beanspruchungsbedingte Besonderheiten berücksichtigt werden sollten, zeigten Autoren, die beschrieben, dass sich bei Läufern die Ausprägung von Flexions- und Extensionsparametern anders darstellte, als beispielsweise bei Radfahrern [8]. Auch im militärsportlichen Kontext wurden Spezifika berichtet [16]. Gut beschrieben ist ebenso die Möglichkeit der Trainingssteuerung des Ausdauertrainings durch Lactat-Leistungsdiagnostik [17]. Auch diese kann somit im Rahmen der Prävention, vor allem zur Detektion von Herz-Kreislauf-Erkrankungen, genutzt werden [18].

Unter Berücksichtigung der Kenntnis des Beanspruchungsprofils der zugewiesenen Soldaten, erschien es somit möglich, spezifische Trainingsempfehlungen aus Messwerten abzuleiten. Auf Grundlage dieser Erkenntnisse etablierten wir eine sportmedizinische Langzeitbetreuung für Soldaten in besonders körperlich beanspruchenden Verwendungen. Im Rahmen der Evaluation sollte anhand anthropometrischer Parameter, Labordaten sowie Messwerten der Lactat-Leistungsdiagnostik und der Rumpfkraft beurteilt werden, ob langfristig ein Erhalt der Leistungsfähigkeit erreicht werden konnte.

## Material und Methoden

### Patienten

In dieser retrospektiven Untersuchung wurden Daten von Soldaten evaluiert, die im Untersuchungszeitraum von 2008 bis 2018 regelmäßig, mindestens aber viermal, in einem zeitlichen Abstand von etwa 18–24 Monaten, im Rahmen einer sportmedizinischen präventiven Untersuchung vorstellig wurden. 443 Soldaten konnten initial identifiziert werden. Bei 256 von ihnen waren 4 Untersuchungszeitpunkte dokumentiert. Bei 115 Soldaten lagen vollständige Datensätze vor. Die Soldaten gehörten einer militärischen Einheit an und waren in einer physisch anspruchsvollen Verwendung eingesetzt, bei der auch eine hohe sportliche Aktivität gefordert war.

Frauen waren im Untersuchungszeitraum in dieser Einheit nicht eingesetzt und konnten somit nicht untersucht werden. Alle Patienten wurden über die wissenschaftliche Auswertung der Daten vorab informiert und erklärten schriftlich ihr Einverständnis. Die Untersuchung erhielt ein positives Votum der zuständigen Ethikkommission (Referenznummer: A2019–0057) und wurde im Deutschen Register Klinischer Studien (DRKS00031820) angemeldet.

### Datenerhebung und Intervention

Im Rahmen der Untersuchungen wurden Alter (in Jahren), Gewicht (in Kilogramm), Größe (in Zentimetern), Taillenumfang (in Zentimetern, gemessen in paralleler Linie zum Boden auf der Hälfte zwischen Crista iliaca und unterem Rippenbogen), Body-Mass-Index in kg/m^2^ und die Waist-to-Height-Ratio (WHtR, dimensionslos) ermittelt. Zusätzlich wurden anamnestische Angaben zum Sportverhalten, Herzfrequenz und Blutdruck in Ruhe im Liegen sowie laborchemische Parameter (Blutbild, ALAT, ASAT, ɣ‑GT, Natrium, Kalium, Kreatinin, Harnstoff, Blutzucker, Cholesterin, Triglyceride, HDL und LDL) erhoben. Die körperliche Leistungsfähigkeit wurde durch sportleistungsdiagnostische Daten mittels Laufbandergometrie, Sprinttest und Daten der isokinetischen Rumpfkraft erhoben. Alle Daten bildeten die Grundlage für eine sportmedizinische Beratung. Dabei wurden auf Basis der Rumpfkraftwerte individuell Hinweise für das Training der Rumpfmuskulatur gegeben und auf Basis der Leistungsdiagnostik auf dem Ergometer wurden Trainingsbereiche für das Ausdauertraining festgelegt.

### Isokinetische Messung der Rumpfkraft

Die Erfassung der Kraft des Rumpfes erfolgte isokinetisch gerätegestützt mittels IsoMed 2000 (D&R Ferstl GmbH, Hemau, Deutschland). Nach vorherigem 10-minütigem Aufwärmen auf einem Fahrradergometer wurden die Messungen durch einen erfahrenen Untersucher durchgeführt. Die Probanden wurden dabei zu maximaler Anstrengung aufgefordert. Die Messung erfolgte im Sitzen mit 90° gebeugtem Hüftgelenk. Bei einer konstanten Winkelgeschwindigkeit von 90°/s mussten die Teilnehmer 10 Wiederholungen Beugung und Streckung im Rumpf mit maximaler Kraft absolvieren. Daraus wurden die Mittelwerte für Drehmoment in Newtonmeter, verrichtete Arbeit in Joule und Leistung in Watt ermittelt sowie das maximale Drehmoment erfasst. Für das maximale Drehmoment, die Arbeit und die Leistung wurden zusätzlich Flexions-Extensions-Verhältnisse berechnet.

### Lactat-Leistungsdiagnostik

Die Erfassung der Ausdauerleistungsfähigkeit erfolgte mittels Laufbandergometrie (Laufband: 70/200; Woodway GmbH, Weil am Rhein; Deutschland) unter stetiger EKG-Kontrolle (Spacelabs Healthcare, Hawthorne, CA, USA). Das Belastungsprotokoll wurde standardisiert mit 8 km/h bei einer Steigung von 1,5° gestartet. Eine Steigerung der Geschwindigkeit um 2 km/h erfolgte alle 3 min. Der Test wurde gemäß international anerkannten Empfehlungen durchgeführt und abgebrochen bei Ausbelastung, Abbruchwunsch des Probanden oder Zeichen der Ischämie im EKG [19]. Ergebnisse abgebrochener Tests wurden nicht mit in die statistische Auswertung aufgenommen. Die Messungen der kapillären Lactat-Konzentration erfolgte jeweils vor Beginn der Belastung, nach jeder Belastungsstufe, sowie während der Erholungsphase. Hierzu wurde mittels Lanzette (Safety Lanzette, Einstichtiefe 1,8 mm, Saarstedt, Nürnbrecht, Deutschland) das Ohrläppchen punktiert und durch manuelle Kompression Blutstropfen gewonnen. Der erste Blutstropfen wurde verworfen und der folgende Blutstropfen in einem Kapillargefäß (heparinisierte End-zu-End-Kapillare, EKF, Magdeburg, Deutschland) gesammelt. Das entnommene Kapillarblut wurde anschließend mittels Spektrometer (Biosen S‑Line: EKF; Magdeburg, Deutschland) analysiert. Die Ergebnisse der Laufbandergometrie wurden anschließend mittels Winlactat (Version V 4.6.3.21, Mesics GmbH, Münster, Deutschland) ausgewertet und grafisch dargestellt, sowie die Parameter Geschwindigkeit (v) und Herzfrequenz (HF) bei 4 mmol/l Lactat und bei maximaler Belastung ermittelt.

### Sprintfähigkeit

Die Teststrecke der Sprintläufe betrug 50 m. Die Probanden durchliefen die Strecke auf einer Indoor-Tartanbahn jeweils 3‑mal nach zwischenzeitlich individuellen Erholungspausen von einigen Minuten. Der Start erfolgte aus dem Stand ohne Startsignal. Die Zeit wurde mittels dreier Lichtschranken gemessen, die jeweils 0,3 m nach der Startlinie, nach 20 m und 50 m platziert waren. Gewertet wurde das jeweils beste Ergebnis und die so ermittelte Zeit in Sekunden in die Auswertung aufgenommen.

### Statistische Auswertung

Die Daten der Stichprobe wurden zunächst deskriptiv ausgewertet, Mittelwerte (MW) und Standardabweichung (SD) bestimmt. Weiterhin wurden die erhobenen Messwerte nach Plausibilitätsprüfung mit SPSS Version 29.0.1.1 (IBM, Armonk, NY, USA) bearbeitet und analysiert. Die Prüfung auf Normalverteilung erfolgte sowohl mittels Shapiro-Wilk-Test als auch optischer Normalverteilungsanalyse mittels Histogrammen und Q‑Q-Plots. Im Falle von Normalverteilung wurde bei den vorliegenden abhängigen Stichproben eine einfaktorielle Varianzanalyse (ANOVA) mit Messwertwiederholung durchgeführt. Als Innersubjektfaktoren wurden dabei die 4 Vorstellungen nach sportmedizinischer Intervention gewählt. Körpergröße und Alter wurden aufgrund ihrer fehlenden Beeinflussbarkeit als Zwischensubjektfaktoren festgelegt. Die Post-hoc-Analysen erfolgten mittels paarweisen Vergleichen nach Bonferroni. Signifikante Unterschiede bestanden bei *p* < 0,05. Anschließend erfolgte für die ermittelten metrischen Werte die Korrelationsanalyse nach Pearson. Der Korrelationskoeffizient (r) zur Darstellung von linearen Zusammenhängen zwischen den gewählten Parametern wurde ermittelt. Das Signifikanzniveau wurde für *p* = 0,05 festgelegt. Die Interpretation der Stärke der Korrelation erfolgte nach Cohen [20]. Ausgewertet wurden die 115 vollständigen Datensätze der Probanden.

## Ergebnisse

### Anthropometrische Messungen

**Abb. 1:**
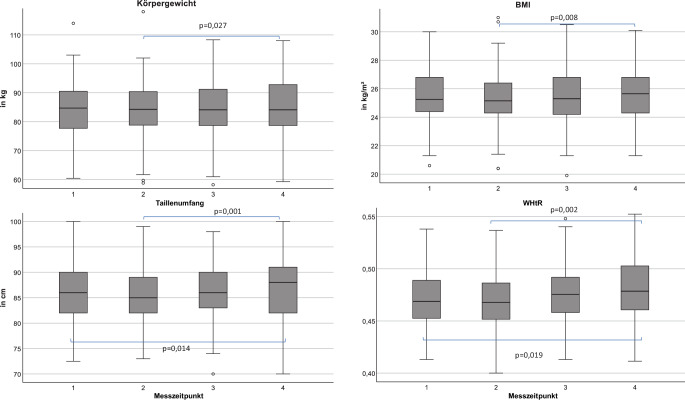
Entwicklung anthropometrischer Werte; Körpergewicht in Kilogramm, Body-Mass-Index (BMI) in kg/m^2^, Taillenumfang in Zentimeter, Waist-to-Hight-Ratio (WHtR, dimensionslos) in der Kohorte im Beobachtungszeitraum

Körpergewicht und BMI nahmen im Beobachtungszeitraum von der zweiten Wiedervorstellung zur vierten Wiedervorstellung zwar signifikant zu (Abb. 1), allerdings war diese Zunahme mit weniger als 1 kg bzw. weniger als 1 kg/m^2^ klinisch nicht relevant. Ebenfalls konnte eine signifikante Zunahme des Taillenumfangs und der WHtR von der zweiten zur dritten bzw. von der zweiten zur vierten Wiedervorstellung beobachtet werden (Abb. 1). Auch hier war die Veränderung mit 1 cm bzw. 0,006 nicht klinisch bedeutsam.

### Sportliche Leistungsfähigkeit

**Abb. 2:**
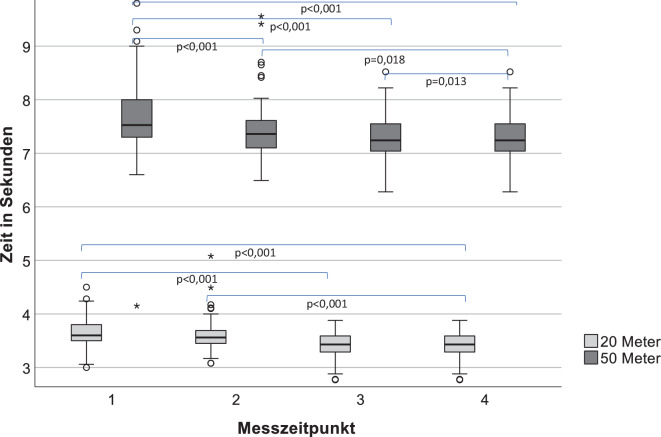
Darstellung der Entwicklung der benötigten Zeiten für eine 20- bzw. 50-m-Sprintstrecke im Beobachtungszeitraum

**Abb. 3:**
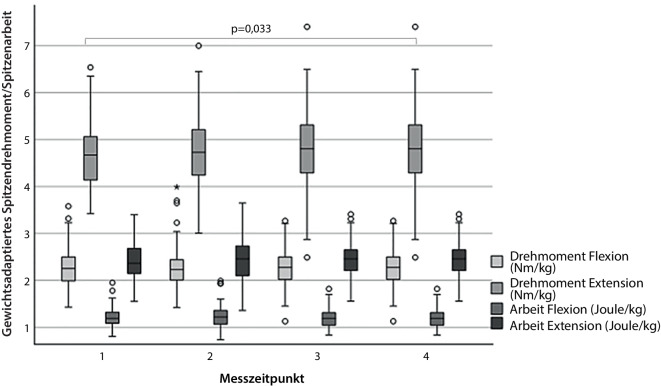
Darstellung der Entwicklung der Rumpfkraft (Arbeit in Flexion und Extension in Joule sowie maximales Drehmoment in Extension und Flexion in Newtonmeter pro Kilogramm) im Beobachtungszeitraum

Im Sprinttest konnte sowohl nach 20 m als auch nach 50 m die benötigte Zeit von Vorstellung zu Vorstellung signifikant reduziert werden (Abb. 2). Der berichtete Trainingsaufwand erhöhte sich von Vorstellung 1 zu Vorstellung 3 signifikant von 4 auf 5 h/Woche (*p* = 0,039) und blieb dann konstant. Dabei wurden anamnestisch sowohl Krafttraining als auch Ausdauertraining jeweils um wöchentlich eine halbe Stunde von Vorstellung 1 zu Vorstellung 3 intensiviert. Diese Veränderung brachte zwar keine statistische Signifikanz, ist aber durchaus von klinischer Bedeutung. In der Korrelationsanalyse zeigte sich, dass eine langsamere Zeit mit höherem BMI (r = 0,185; *p* = 0,048) und höherer WHtR (r = 0,219; *p* = 0,019) verbunden war. Eine schnellere Zeit korrelierte dabei mit mehr Krafttraining (r = −0,185; *p* = 0,049). Die errechnete Geschwindigkeit bei 4 mM Lactat im Rahmen der Ergometrie lag bei allen Messungen konstant bei 13,2 km/h (± 1,3) und die erreichte maximale Geschwindigkeit lag konstant bei 16,3 km/h (± 0,7). Sie korrelierte dabei signifikant negativ mit den anthropometrischen Parametern BMI (r = −0,513; *p* < 0,001), Taillenumfang (r = −0,469; *p* < 0,001) und WHtR (r = −0,537; *p* < 0,001) sowie positiv mit maximalem Drehmoment in Flexion (r = 0,2; *p* = 0,033) und Extension (r = 0,23; *p* = 0,014). Das maximale Extensionsdrehmoment im Rahmen der isokinetischen Rumpfkraftmessung konnte von 4,6 auf 4,8 Nm/kg (*p* = 0,033) bei der 3. Vorstellung signifikant verbessert werden (Abb. 3). Im Anschluss konnte das Niveau gehalten werden. Im Bereich der Rumpfflexion kam es zu keiner signifikanten Veränderung und das Niveau konnte bei 2,27 Nm/kg konstant gehalten werden (Abb. 3). Das Flexions-Extensions-Verhältnis lag konstant bei 49 % (± 12).

### Sonstige Parameter

**Abb. 4:**
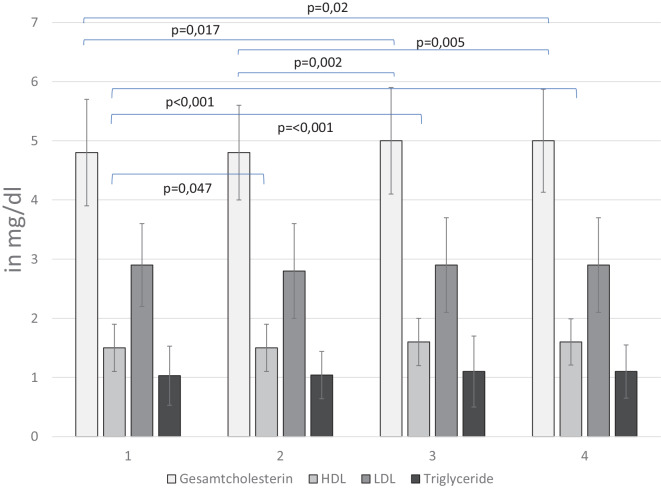
Darstellung der Entwicklung der Fettstoffwechselparameter (Gesamtcholesterin, HDL[„high density lipoprotein“]-Cholesterin, LDL[„low density lipoprotein“]-Cholesterin und der Triglyceride) im Beobachtungszeitraum

Die Ruheherzfrequenz blieb konstant bei 60 (± 12) Schlägen/Minute. Der systolische Blutdruck in Ruhe verringerte sich signifikant von 124 (± 10) auf 120 (± 10) mm Hg zur 3. Vorstellung (*p* = 0,017) und blieb dann konstant, während der diastolische Blutdruck konstant bei 77 (± 7) mm Hg beobachtet wurde. Alle ermittelten Laborparameter verblieben auf gleichbleibendem Niveau im Normbereich. Lediglich der Parameter Gesamtcholesterin erhöhte sich von Messung 1 zu Messung 3 und 4 signifikant (Abb. 4). Während sich Triglyceride und LDL-Cholesterin nicht signifikant veränderten, kam es parallel ebenfalls zu einer signifikanten Erhöhung des HDL-Cholesterins (Abb. 4). HDL-Cholesterin korrelierte dabei signifikant negativ mit den anthropometrischen Parametern Gewicht (r = −0,195; *p* = 0,038), Taillenumfang (r = −0,26; *p* = 0,005), BMI (r = −0,245; *p* = 0,009) und WHtR (r = −0,259; *p* = 0,005). Ein Zusammenhang mit Trainingshäufigkeit, Geschwindigkeit auf dem Laufband oder der Entwicklung der Rumpfkraft ließ sich statistisch nicht nachweisen.

## Diskussion

Die Evaluierung der sportmedizinischen Langzeitbetreuung hinsichtlich des Erhalts der körperlichen Leistungsfähigkeit und der Rumpfkraft sollte mittels anthropometrischer Parameter, Laboruntersuchungen, Lactat-Leistungsdiagnostik, Sprinttest und Ermittlung von Rumpfflexions- und -extensionskraft erfolgen. Bei Betrachtung der Werte Körpergewicht und Taillenumfang sowie, der damit zu berechnenden Indices BMI und WHtR, zeigte sich ein konstanter Verlauf, der im Rahmen der 4. Wiedervorstellung jeweils zwar signifikant erhöht war, bei Betrachtung des Ausmaßes von etwa 1 kg bzw. 1 cm im Vergleich zur Ausgangsmessung aber als wenig klinisch relevant bewertet werden muss. Die Erhöhung des BMI könnte auch mit einer Zunahme der Muskelmasse erklärt werden [21]. Eine Zunahme des Taillenumfangs und damit der WHtR ist eher auf das abdominale Fett zu beziehen [22]. Letztere Parameter erscheinen daher eher geeignet im Rahmen der Prävention ungünstige Veränderungen anzuzeigen, da insbesondere für die relative Fettmasse ein stärkerer Einfluss auf die Mortalität nachgewiesen wurde [23].

Soldaten zeigten eine Verbesserung der Sprintfähigkeit bei Zunahme der Rumpfkraft

Da sich die anthropometrischen Parameter nicht relevant verändert haben, ist diesbezüglich die Prävention erfolgreich gewesen. Das Ausgangsniveau lag in einem adäquaten Bereich und die Soldaten konnten eine bedeutsame Gewichtszunahme vermeiden, wie sie im mittleren Lebensalter häufig beobachtet werden kann, [24]. Ebenso konnte das Niveau der maximalen Geschwindigkeit im Rahmen der Lactat-Leistungsdiagnostik über den etwa 6‑jährigen Beobachtungszeitraum konstant gehalten werden. Die Sprintfähigkeit konnte sogar verbessert werden. Dies ging einher mit der Beobachtung einer stärkeren Rumpfextensionskraft, wie es zuvor bei Läufern berichtet wurde [8].

Auch eine frühere Querschnittsuntersuchung bei Soldaten zeigte eine Verbesserung der Sprintfähigkeit bei Zunahme der Rumpfkraft [25]. Dies ist möglicherweise auch Resultat der Trainingsberatung, die an einer relevanten Adaptierung der Trainingsgewohnheiten beteiligt war. Allerdings können die Ursachen durch verschiedenste Faktoren mitbeeinflusst werden, die im Rahmen der statistischen Auswertung nicht bewertet wurden (z. B. Ernährungsverhalten). Bei einer im Wesentlichen normalgewichtigen Kohorte hatten die anthropometrischen Parameter keinen relevanten Einfluss auf die Sprintfähigkeit. Auch ein Einfluss auf die Entwicklung der Rumpfkraft ist unwahrscheinlich [26]. Die Rumpfkraft erlaubt zudem Rückschlüsse auf die körperliche Leistungsfähigkeit generell [8].

Somit kann auch unter Berücksichtigung aller leistungsmedizinischen Daten die Prävention des Leistungsverlustes in der beobachteten Kohorte angenommen werden, da auch die Leistungsfähigkeit im Rahmen der Laufbandergometrie erhalten werden konnte.

Im Rahmen der Untersuchungen wurden weitere Parameter (ASAT, ALAT, Amylase, Blutbild, Elektrolyte, Kreatinin, Harnstoff, Kreatinkinase, C‑reaktives Protein) bestimmt. Ebenso wie bei Ruheherzfrequenz und Blutdruck zeigten sich Normalwerte und keine signifikanten Veränderungen. Lediglich der systolische Blutdruck konnte signifikant reduziert werden. Sowohl Ausgangs- als auch Kontrollwerte lagen immer im Bereich, für den keine Risikoerhöhung bezüglich Herz-Kreislauf-Schäden zu erwarten waren [27].

Die Erhöhung des Gesamtcholesterins, welche auf die Erhöhung des HDL-Cholesterins zurückzuführen war, bedeutete eine positive Entwicklung, da ein Anstieg des HDL, im Ausmaß der hier erreichten Werte, positiv bezüglich des Auftretens von Herz-Kreislauf-Erkrankungen bewertet werden kann und auch in Folge einer Trainingsintensivierung beobachtet werden konnte [28, 29].

Limitationen der Untersuchung sind neben dem retrospektiven Charakter auch eine fehlende Korrelationsmöglichkeit mit dem Auftreten von Krankheitsereignissen oder Ausfallzeiten, da deren Dokumentation nicht in unserem Zentrum erfolgte, sondern Aufgabe des Truppenarztes am jeweiligen Heimatstandort war. Es sollte Ziel prospektiver Untersuchungen sein, zu prüfen, inwieweit in der Klientel körperlich stark beanspruchter Soldaten der Zustand der körperlichen Leistungsfähigkeit mit dem Auftreten von Erkrankungen korreliert und ob sich neben dem Erfolg beim Erhalt der körperlichen Leistungsfähigkeit auch ein Erfolg bei der Vermeidung des Auftretens von Ausfallzeiten zeigt. Eine weitere Limitation dieser Studie stellt die untersuchte Personengruppe an sich dar. Um für eine Verwendung in einer Einheit ausgewählt zu werden, die im Rahmen der Studie als Personengruppe untersucht wurde, wurde das Vorliegen gesundheitlicher Einschränkungen bereits bei der Rekrutierung ausgeschlossen. Frauen konnten nicht ausgewählt werden. Rückschlüsse auf eine Bedeutung für andere Bevölkerungsgruppen sind daher nur begrenzt möglich.

Aus orthopädischer Sicht wäre die Vermeidung von Rückenschmerzen bedeutsam, wobei ein gutes Flexions-Extensions-Verhältnis unter 69,2 % im Bereich der elektromyografisch ermittelten Muskelaktivität als protektiv angesehen wird [30]. Im Bereich der Rumpfkraft wird ein Flexions-Extensions-Verhältnis zwischen 50 und 90 % in Abhängigkeit vom sportlichen Anspruch als Normalwert angesehen, wobei sportlich ambitionierte Personen eher einen Zielwert zwischen 50 und 70 % anstreben sollten [31]. Als Diagnostikum können die Werte der Rumpfkraft ebenfalls herangezogen werden [31, 32], wobei es insbesondere für das Verhältnis von Flexions- zu Extensionskraft Erkenntnisse dahingehend gibt, dass sich die Messwerte zwischen beschwerdefreien Probanden und Patienten mit funktionellen Rückenschmerzen unterscheiden [33]. Das Kraftverhältnis von Flexion zu Extension lag in dieser Kohorte bei 49 % und somit im unteren angestrebten Zielbereich [16]. Bei ausreichender Flexionskraft war aufgrund der spezifischen körperlichen Belastungen (z. B. Tragen von Lasten, Fallschirmspringen) mit einer ausgeprägteren Extensionskraft zu rechnen, weshalb die Ratio, wie erwartet, hier eher im niedrig normalen Bereich ermittelt wurde. Das wurde auch in der Trainingsberatung gewürdigt, um ein weiteres Absinken des Wertes zu vermeiden [16].

Die gewonnenen Erkenntnisse zeigen, unter Berücksichtigung der bestehenden Literatur, dass die Rumpfkraft ein wesentlicher Parameter für die Beurteilung der körperlichen Leistungsfähigkeit bei militärischem Personal ist und Potenzial hat möglicherweise auch als Anzeiger für die Wahrscheinlichkeit des Auftretens von Ausfallzeiten zu fungieren. Diesbezüglich könnte ein Screening bei beanspruchten Personen sinnvoll sein, um Rückschlüsse auf die Vermeidung von Krankheitsereignissen zu erhalten. Prospektive Untersuchungen sollten dazu durchgeführt werden.

## Fazit für die Praxis

- Eine kombinierte Kontrolluntersuchung mit Beachtung von Rumpfkraft- und Ausdauerparametern sowie die Beobachtung möglicher Risikofaktoren bei hochbelasteten Soldaten ist sinnvoll.
- Eine Trainingsberatung unterstützt beim Erhalt/Ausbau der Leistungsfähigkeit von hochbelasteten Soldaten.
- Die Lactat-Leistungsdiagnostik bei hochbelasteten Soldaten ist ein wertvoller Bestandteil im Rahmen der präventiven Untersuchung und gibt Hinweise auf den Verlauf der Leistungsfähigkeit und die Möglichkeit für Empfehlungen zur Optimierung des Trainings.
- Die isokinetische Rumpfkraftmessung stellt bei dieser Kohorte einen wichtigen Bestandteil der präventiven Untersuchungen dar und liefert Hinweise auf Risikokonstellationen für das Auftreten von z. B. Rückenschmerzen, kann aber auch als Kontrollparameter für die Umsetzung von Trainingsempfehlungen dienen.
- Der Zusammenhang zwischen Erhalt der Leistungsfähigkeit und der Verringerung von Krankheitslast in dieser Kohorte muss noch untersucht werden.

## Abkürzungen

ALAT: Alanin-Aminotransferase
ASAT: Aspartat-Aminotransferase
BMI: Body-Mass-Index
ɣ GT: γ‑Glutamyltransferasen
HDL: „High-density lipoprotein“
HF: Herzfrequenz
LDL: „Low-density lipoprotein“
MW: Mittelwert
SD: Standardabweichung
WHtR: Waist-to-Height-Ratio
